# Comparative Metagenomic Analysis of Human Gut Microbiome Composition Using Two Different Bioinformatic Pipelines

**DOI:** 10.1155/2014/325340

**Published:** 2014-02-25

**Authors:** Valeria D'Argenio, Giorgio Casaburi, Vincenza Precone, Francesco Salvatore

**Affiliations:** ^1^CEINGE-Biotecnologie Avanzate, Via G. Salvatore, 80145 Naples, Italy; ^2^Department of Molecular Medicine and Medical Biotechnologies, University of Naples Federico II, Via S. Pansini, 80131 Naples, Italy; ^3^IRCCS-Fondazione SDN, Via Gianturco, 80143 Naples, Italy

## Abstract

Technological advances in next-generation sequencing-based approaches have greatly impacted the analysis of microbial community composition. In particular, 16S rRNA-based methods have been widely used to analyze the whole set of bacteria present in a target environment. As a consequence, several specific bioinformatic pipelines have been developed to manage these data. MetaGenome Rapid Annotation using Subsystem Technology (MG-RAST) and Quantitative Insights Into Microbial Ecology (QIIME) are two freely available tools for metagenomic analyses that have been used in a wide range of studies. Here, we report the comparative analysis of the same dataset with both QIIME and MG-RAST in order to evaluate their accuracy in taxonomic assignment and in diversity analysis. We found that taxonomic assignment was more accurate with QIIME which, at family level, assigned a significantly higher number of reads. Thus, QIIME generated a more accurate BIOM file, which in turn improved the diversity analysis output. Finally, although informatics skills are needed to install QIIME, it offers a wide range of metrics that are useful for downstream applications and, not less important, it is not dependent on server times.

## 1. Introduction

Microbes play an important role in virtually all ecosystems ranging from those in the sea or the soil [1, 2] to those in human body environments like the skin or the gut [3–5]. The link with human body environments generated many studies of microbial community composition designed to assess its role in various metabolic pathways and to determine whether it is involved in inducing and/or preventing specific pathological conditions. Such investigations could help to clarify the pathogenesis of specific diseases and could also lead to novel disease-markers and/or to the development of novel therapeutic strategies. To date, several human diseases have been significantly correlated with dysbiosis of specific microbial communities [6–9].

Thanks to technological improvements in sequencing methods, virtually all the microbes from a given environment can be analyzed in a single run, avoiding cultivation steps. In particular, procedures based on 16S rRNA next-generation sequencing, which allow the high throughput microbial identification within a specific metagenome, represent a powerful means to investigate the composition and the biodiversity of microbial communities [10]. The enormous amount of next-generation metagenomic data generated by such procedures necessitates bioinformatic tools able to analyze them. In fact, an accurate taxonomic assignment of each microbe in a target environment is required to evaluate the structure, the biodiversity, the richness, and the role of the community resident in a given environment [11, 12].

MetaGenome Rapid Annotation using Subsystem Technology (MG-RAST) is a freely available (http://metagenomics.nmpdr.org), fully automated system able to process metagenome sequence data by performing sequence alignment, sequence functional and phylogenetic assignments, and comparative metagenomics [13]. Quantitative Insights Into Microbial Ecology (QIIME) is an open-source software pipeline (http://qiime.sourceforge.net/) able to perform, starting from raw sequence data, a wide range of analyses on microbial communities, that is, sequence alignment, identification of operational taxonomic units (OTUs), elaboration of phylogenetic trees, and phylogenetic and taxon-based analysis of diversity within and between samples [14]. Both tools have been successfully used to analyze a large number of metagenomic 16S ribosomal RNA datasets by assessing their ability in the management of these kinds of data [15, 16].

We have performed a comparative bioinformatic analysis of the same dataset using both QIIME and MG-RAST to evaluate their accuracy in taxonomic assignment. Here, we report the efficacy of these two well established methods in assigning sequence reads to microbes at different phylogenetic levels and in analyzing the diversity and richness of microbial communities.

## 2. Materials and Methods

### 2.1. 16S rRNA Sequence Dataset

We constructed a dataset containing the 16S rRNA sequence data obtained from the analysis of the ileum mucosa samples of four unrelated children: two patients with inflammatory bowel disease and two sex- and age-matched healthy controls. The next generation sequencing evaluation of their gut microbial communities was carried out as previously described [8].

### 2.2. Bioinformatics Analysis

#### 2.2.1. Preanalysis Step

The following parameters were set for both QIIME and MG-RAST: (i) a minimum average quality Phred score of 25 allowed in reads; (ii) a minimum and maximum sequence length in the range of 200–1000 nucleotides; and (iii) a maximum number of ambiguous bases and length of homopolymers equal to 6. In addition, to be as stringent as possible, we did not allow any primer mismatches (setting the parameter “primer mismatches” = 0) and allowed only a 1.5 maximum number of errors in barcodes.

#### 2.2.2. 16S rRNAs Detection, Clustering, and Identification

16S bacterial rRNAs identification was performed by the two tools using two distinct strategies. MG-RAST computes the 16S rRNAs search with the Blast-Like-Alignment Tool (BLAT) [17] against a reduced rRNAs database. This reduced database is obtained from a 90% identity clustered version of the SILVA [18] database and is used to increase the rate of identification of the sequences similar to specific rRNAs,thereby reducing the computing time. The selected rRNA reads are then clustered at 97% identity by picking the longest sequence within each cluster as representative of that cluster. An additional similarity search with BLAT is then performed using only the obtained representative cluster-sequences against different 16S rRNA databases which can be selected by the user (see *Taxonomic classification* and Table 1). We used MG-RAST default clustering parameters within the BLAT algorithm.

In QIIME, the 16S rRNAs detection is performed with an OTU-picking approach. The OTU-picking procedure consists in assigning sequences to OTUs by clustering the sequences on the basis of a threshold that the user may modify. When a sequence shows a similarity level near or above the chosen threshold, it is taken in a sequence collection that represents the presence of a taxonomic unit. QIIME implements several clustering methods to perform this operation. We used the default clustering algorithm UCLUST [19], which creates sequence clusters based on percent identity (default identity = 97%). After the OTU picking step, the representative sequence for each OTU, namely, the most abundant sequences in that OTU, is chosen for subsequent analyses in order to reduce the computational power and the analysis time, without losing the frequency information.

#### 2.2.3. Taxonomic Classification

In MG-RAST, the taxonomic classification was performed with BLAT [17] and, for comparison purposes, we selected, among the available 16S rRNA databases, the Greengenes database (2012 release, available at http://greengenes.lbl.gov/) [20], setting the *Max e-Value Cutoff* to 1 × 10^5^ and the *Min% Identity Cutoff* to 80% (Table 1). Reads assigned to the *Bacteria* root but not attaining the threshold at the chosen taxonomic level fell in the category “Unclassified”, while sequences not assigned to the *Bacteria* root were classified as “No Hits”. To compare the power of taxonomic assignment of the two pipelines, we extracted the obtained results at amily level. After taxonomic assignment, MG-RAST generates a web page for results visualization and handling, and it can also generate a Biological Observation Matrix (BIOM) file useful to transfer the obtained data to other tools for comparison purposes [21].

QIIME can perform the taxonomy assignment using different methods (Table 1) [22]. We used the Ribosomal Database Project (RDP) classifier 2.2 [23] against the Greengenes database (2012 release, available at http://greengenes.lbl.gov/) [20] using the same thresholds we used for MG-RAST. After taxonomic assignment, QIIME generates a BIOM file that can be used for a wide range of analyses [21].

#### 2.2.4. Diversity Analysis

To obtain an overall diversity analysis for subsequent comparative and statistical evaluations, we merged the BIOM tables generated by both MG-RAST and QIIME in a unique *biom table*, using a script included in QIIME (*merge_otu_tables.py*). Thus, we obtained a unique matrix table that reports all the taxonomic assignments and their frequencies obtained by each of the two tools. Subsequently, the diversity analysis was computed on the merged *biom table *using the related scripts included in QIIME.

QIIME alpha diversity analysis script (*alpha_rarefaction.py*) performs the rarefaction analysis by subsampling the OTUs *biom table *on the basis of a minimum rarefaction depth value that is chosen by the user depending on the minimum number of sequences/sample obtained. For our subset, this value was 1,195. Then, using different metrics, the alpha diversity was computed for each rarefied *OTUs table* (Table 2). We used three “non-phylogeny-based” metrics, namely, the observed species, chao 1 [24], and the Shannon index [25]. Finally, all the results obtained from each rarefied *OTUs table* are joined in three global alpha diversity measures, one for each metric used, and converted in  .*html* plots in order to handle and visualize the data.

QIIME beta diversity analysis script (*beta_diversity_through_plots.py*), after the rarefaction evaluation (this step corresponds to the first step of the alpha diversity workflow), computes the beta diversity on the rarefied *OTUs tables *using different metrics (Table 2). We used the Bray-Curtismetric [26]. Finally the script uses the obtained distance metric to compute the Principal Coordinate Analysis (PCoA) and to convert it into plots for results visualization.

#### 2.2.5. Data Comparison and Statistical Analysis

We computed the Analysis of Variance (ANOVA) [27] and the *G*-test [28] using a Bonferroni correction [29] to determine the statistical significance of each taxon assigned by the two tools. In addition, we computed a Pearson Correlation [29] between the two datasets to correlate the taxa identified at family level. All these tests are available in QIIME using a *biom table* file as input (Table 2). We performed a nonparametric test [30] and the ANOSIM [31] and ADONIS tests [32, 33] to determine the statistical significance related to the diversity analysis.

## 3. Results

The 16S rRNA next-generation sequencing run produced 48,545 raw sequences belonging to the 4 samples. We used those sequences as input in both the MG-RAST and QIIME analysis workflows. The time required to complete the analysis was about 10 days for MG-RAST and less than 2 hours for QIIME. This big time difference in both methods can be explained considering the following aspects. MG-RAST is a web server, therefore the analysis time depends on the number of projects simultaneously submitted by different users and on the priority level selected. In particular, for our data we selected the “Lowest Priority” level to keep them private and, of course, this setting requires a longer time. On the contrary, QIIME is an installable software package; in this case, the analysis time depends just on the amount of user data and on his bioinformatic ability. In our dataset, we had just four samples and a skilled user. After the filtering step, we obtained 35,232 and 38,813 postfiltering reads for MG-RAST and QIIME, respectively (Table 1).

**Figure 1:**
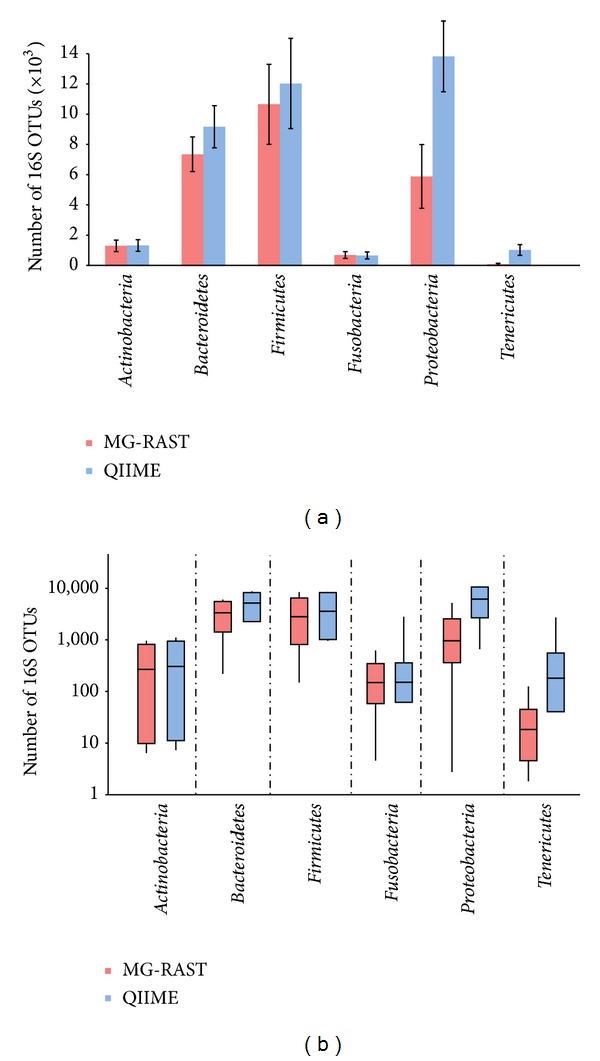
Phyla distribution in the studied dataset as computed by each tool. Comparison of the bacterial phyla identified by MG-RAST and QIIME related to the numbers (a) and to the Log10 scale (b) of the 16S OTUs identified. Error bars indicate standard deviations of the mean. Box plots (b) show phyla distribution in the two groups; the 25th percentile, median, and 75th percentiles are reported by horizontal lines. Whiskers represent minimum and maximum values (b).

Both tools identified 6 main Phyla within the root *Bacteria: Actinobacteria, Bacteroidetes, Firmicutes, Fusobacteria, Proteobacteria,* and *Tenericutes.* The number of 16S OTUs assigned to each Phylum is shown in Figure 1(a). Despite some differences between MG-RAST and QIIME, particularly in the *Proteobacteria*, there were no statistical differences at Phylum level. The observed differences are due to a single sample differently assigned by the two tools but normalized during statistical analysis (ANOVA). The same 16S OTUs distribution at Phylum level is reported also in Log10 scale, showing the 25th, 50^th^, and 75th percentiles. Minimum and maximum values for each Phylum, as computed by the tools, are shown as whiskers (Figure 1(b)).

**Figure 2:**
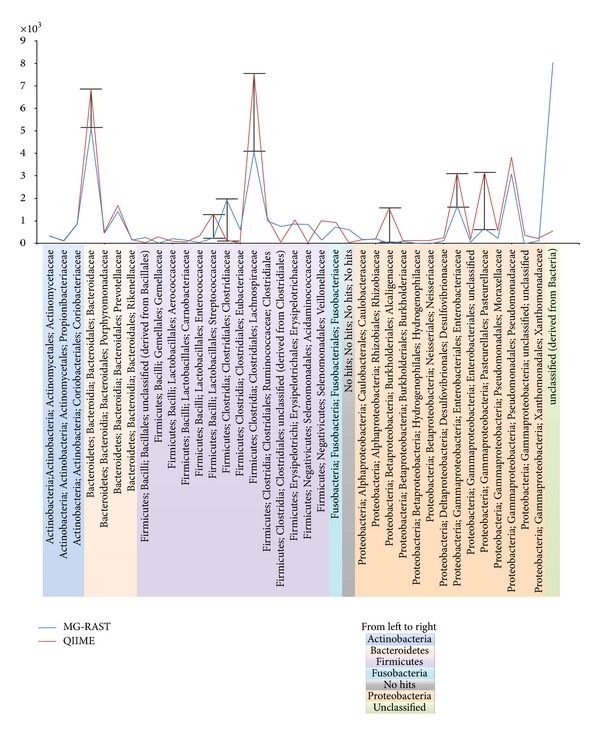
Family level taxonomic composition correlated with the number of OTUs identified by Mg-Rast and QIIme. Two distinct trends are shown, respectively, for both the tools. Different colors represent different Phyla that belong to the identified families. Qiime reported higher general values for each family compared to Mg-Rast. Seven families were identified with a widely different score (>1000 sequences, indicated by bars): *Bacteroidaceae *(belongs to *Bacteroidetes *Phylum, in yellow); *Streptococcaceae, Clostridiaceae,* and *Lachnospiraceae* (belong to *Firmicutes* Philum, in purple);* Alcaligenaceae*, *Enterobacteriaceae, *and *Pasteurellaceae *(belong to *Protecobacteria* Philum, in orange). No hits field (in gray) represents those sequences who were not assigned to the *Bacteria* root (605 for Mg-Rast and 12 for QIIme). Unclassified field (in green) represents those sequences who belong to the *Bacteria* root but both the tools were unable to identify precise taxonomy.

At deeper phylogenetic levels, we found some interesting differences in the taxonomic assignment between MG-RAST and QIIME. In particular, 70 distinct bacteria Families were identified by MG-RAST and 123 by QIIME, while when considering only Families with more than 100 sequences, 27 and 30 distinct bacteria Families were identified by MG-RAST and QIIME, respectively (Table 1). The taxonomic composition of our dataset, reported at family level according to the number of 16S rRNAs identified by MG-RAST and QIIME, showed two distinct trends for the two tools (Figure 2). Globally, QIIME assigned higher number of reads to each family than did MG-RAST. In detail, 7 Families were identified with a widely different score (Δ > |1000| Sequences): *Bacteroidaceae (Bacteroidetes phylum); Streptococcaceae, Clostridiaceae,* and *Lachnospiraceae (Firmicutes phylum); Alcaligenaceae, Enterobacteriaceae,* and *Pasteurellaceae (Proteobacteria phylum)*. Neither tool was able to assign some of the sequences to the *Bacteria* root: 605 “No Hits” sequences for MG-RAST and 12 for QIIME. The sequences assigned to the *Bacteria* root, but with no taxonomical assignment at family level, were reported as “Unclassified”; those sequences were 8,022 for MG-RAST and 525 for QIIME, which was the greatest difference found between the two tools (Figure 2).

**Figure 3:**
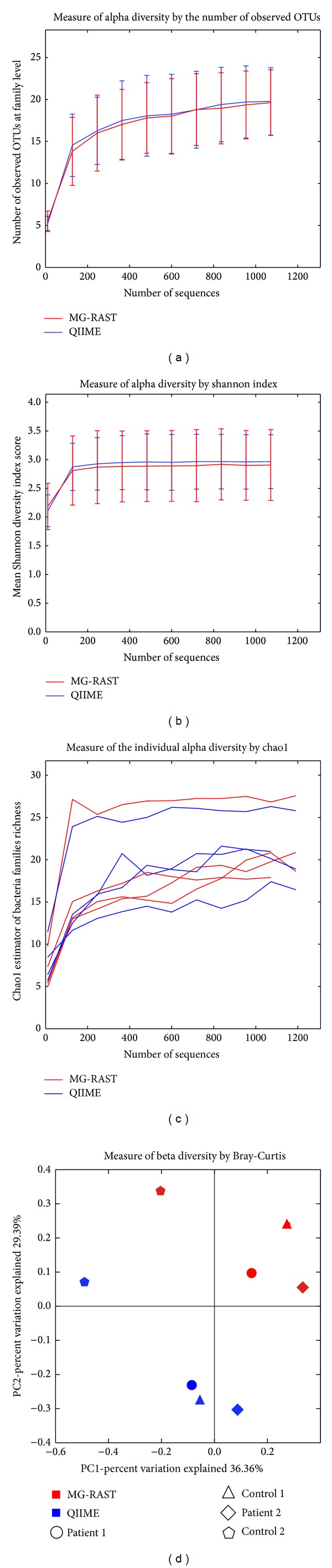
Diversity analysis. Alpha diversity of the identified 16S rRNAs OTUs with MG-RAST and QIIME shows no significant variation between the two tools as measured using the observed species method (a) and Shannon index average scores (b). Also the single rarefaction curves obtained for each sample, as computed by the Chao1 estimator, show similar trends with the two tools (c). Beta diversity analysis among samples was carried out according to the Bray-Curtis metric; the same sample analyzed with the two tools appears to be distant, thus indicating individual differences [*P* = 0.028, *R*2 = 0.3; ADONIS] (d).

Figure 3 shows differences in the diversity analysis carried out by MG-RAST and QIIME. Neither the alpha diversity measured as 16S rRNAs observed OTUs at family level (Figure 3(a)) nor the mean Shannon index score (Figure 3(b)) differed significantly between MG-RAST and QIIME. Similarly, the single rarefaction curves, computed for each sample by Chao1 richness estimator, showed similar trends/sample with the two tools (Figure 3(c)). On the contrary, beta diversity computed with the Bray-Curtis metric showed a significant difference (*P* = 0.028, *R*2 = 0.3; ADONIS) in the same samples analyzed in duplicate by the two tools (Figure 3(d)).

## 4. Discussion

The aim of this work was to compare the efficiency of the bioinformatic analysis of 16S rRNA next-generation sequencing-based data performed by QIIME and MG-RAST, which are the most frequently cited tools in the context of metagenomic analysis.

We first evaluated the accessibility and ease-of-use of the two tools. MG-RAST is a web platform for automated analysis, while QIIME is an open-source software package. In the former case, the analysis depends on the times of the MG-RAST server and on its data uploading limits. The user has to submit the raw data to the MG-RAST server specifying if the data is private (visible only to the submitter) or public (data will be shared with all MG-RAST users). MG-RAST provides, associated with a priority queue, five different options with different times of analysis. For scientific research purposes, we choose the “Lowest Priority” (data will remain private) option. The time required by the MG-RAST server to complete the analysis is related to the number of jobs submitted by all MG-RAST users in the analysis queue and to the priority level selected. Sometimes this may not be compatible with the researcher's needs.

QIIME is completely installable and users can start their analysis as soon as the installation is complete (http://qiime.org/install/install.html). However, installation requires some basic informatic knowledge, since several dependencies must be installed separately to use the complete QIIME analysis pipeline. To counteract this limit, QIIME offers various options that can be downloaded free of charge. Once QIIME and all its dependencies are installed, users can start the analysis pipeline. The time necessary to complete installation depends on several factors, mainly the amount of data, the chosen pipeline, and the user's bioinformatic skills.

To minimize differences between MG-RAST and QIIME, we selected the same parameters for the preliminary analysis. This step includes quality filtering, primer detections, and read demultiplexing. MG-RAST provides a preanalysis step, while QIIME integrates the data in a script (*split_libraries.py*). For the preanalysis filtering step, both tools require a metadata mapping file in which the user must provide at least the following information: (i) sample ID and barcode; (ii) primer sequences used for the library construction; and (iii) one or more description columns containing metadata information related to the sample. We included the following additional metadata information: age, sex, and treatment type (patient-control). The mapping file must have a specific format to be accepted by both tools; this step may be complex and result in delays before the analysis workflow can even start. To overcome this drawback, MG-RAST provides a template mapping file that users can edit and modify with their own data, while QIIME includes a script (*check_id_map.py*) that checks the mapping file, identifies errors, and indicates how to solve them.

Identification of 16S rRNAs from a set of quality-filtered sequences can be carried out by MG-RAST only with a limited pipeline (see Section 2.2.2), while QIIME provides three high-level protocols that belong to the OTUs picking procedure: *de novo*, closed-reference, and open-reference OTUs picking. We choose the *de novo* OTUs picking method since the dataset was small and in order not to lose any reads.

Taxonomic assignment can be performed by MG-RAST only with BLAT [17], while several algorithms are available in the QIIME pipeline [34, 35] (Table 1). MG-RAST by default allows the direct use of several 16s rRNA databases (LSU, SSU, M5RNA, RDP, and Greengenes) [36], but it is not possible to use a custom database. By default, QIIME performs the taxonomy assignment against the Greengenes database, but users may supply a custom database which is made compatible with the assignment algorithm (Table 1). In our dataset, both tools were able to detect 6 different Phyla with a similar identity. Interestingly, there were statistically significant differences in the taxonomic identification at family level. In particular, QIIME more accurately assigned reads to the different families while a lower number of reads were assigned to the categories “No Hits” and “Unclassified” (Figure 2).

After the taxonomic assignment, which gives a picture of the microbial community composition, typically a metagenomic analysis pipeline continues to evaluate the microbial diversity both as alpha diversity (quantitative global diversity within a sample) and as beta diversity (qualitative diversity between a collection of samples) [37]. These parameters are useful to estimate community richness and to establish the degree of similarity of the microbial composition among samples. We obtained similar results with MG-RAST and QIIME in terms of alpha diversity measured with different metrics. However, at beta diversity analysis, different values were assigned to the same subject depending on the tool used for the analysis, even though they were obtained with the same metric (Bray-Curtis). Therefore, it is feasible that this discrepancy results from differences in 16S rRNAs identification and taxonomic assignment. In fact, since QIIME results in a higher accuracy in reads assignment (a lower rate of “No Hits” and “Unclassified”) this is converted into a more complete BIOM file, which is the matrix used for diversity evaluation.  Table 3 summarizes the main features of the two tools.

## 5. Conclusions

**Figure 4:**
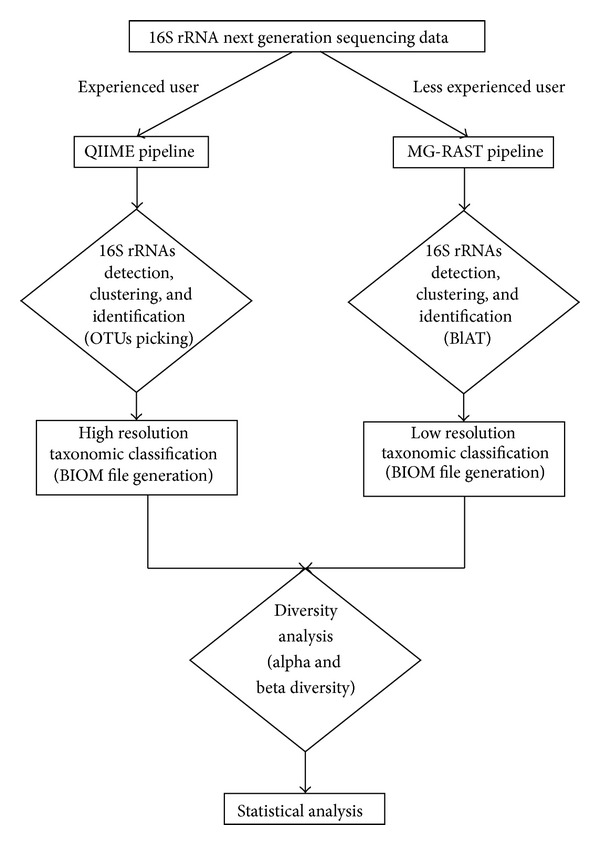
Analysis flowchart. The comparative metagenomic analysis of the gut microbiome composition in the same subjects using both MG-RAST and QIIME pipelines highlighted that better performances can be obtained by QIIME. Since MG-RAST is easier to use it could be useful for first-time users.

We successfully carried out the comparative metagenomic analysis of the gut microbiome composition in the same subjects using both MG-RAST and QIIME pipelines. Our results showed that the QIIME tool provides a more accurate taxonomic identification which is crucial for the subsequent diversity analysis. In addition, being freely downloadable, it does not depend on server times. Finally, QIIME integrates the BIOM file directly in its pipeline and this option is useful for a wide range of downstream analyses and also speeds up the entire workflow. Less experienced operators, however, may find MG-RAST easier to use than QIIME. Therefore, keeping in mind all the abovementioned features, we suggest that MG-RAST could be useful for first-time-users to familiarize with metagenomic analysis output and criticisms. Upgraded versions of QIIME will follow in the next year, including even more features especially a Graphical User Interface (GUI), that will help non-computer-skilled people to easily analyze their data.  Figure 4 summarizes the proposed flowchart.

## Figures and Tables

**Table 1:** Summary of the taxonomic assignment data and of the algorithms available to obtain them in the MG-RAST and QIIME tools.

	Postfiltering reads*	Total distinct bacteria Families^§^	Bacteria families with >100 sequences^†^	Available taxonomic assignment algorithms^‡^	Default available 16S rRNA databases^±^
MG-RAST	35,232	70	27	BLAT	Greengenes, LSU, SSU, M5RNA, RDP, no custom databases

QIIME	38,813	123	30	Rdp, *Blast, Mothur, Rtax *	Greengenes, custom databases

*Number of reads obtained after the quality filtering step by the two bioinformatic pipelines; ^§^number of distinct bacteria Families identified, as total number and ^†^as the most represented Families with more than 100 sequences; ^‡^taxonomy assignment algorithms available for both tools; ^±^16S rRNA databases available for both tools.

**Table 2:** Overview of all diversity analysis metrics and statistical tests available in MG-RAST and QIIME.

	Alpha diversity metrics*	Beta diversity metrics^§^	Statistical tests^†^
MG-RAST	Shannon	Bray-Curtis	Unpaired *t* test ANOVA Mann-Whitney test Kruskal-Wallis test

	*Nonphylogeny based metrics *	*Non-phylogeny based metrics *	
QIIME	berger_parker_d brillouin_d chao1 chao1_confidence dominance doubles equitability fisher_alpha gini index goods coverage heip_e kempton_taylor_q margalef mcintosh_d mcintosh_e menhinick michaelis_menten_fit observed_species osd robbins Shannon simpson (1-Dominance) simpson_reciprocal (1/Dominance) simpson_e singles strong	abund_jaccard binary_chisq binary_chord binary_euclidean binary_hamming binary_jaccard binary_lennon binary_ochiai binary_otu_gain binary_pearson binary_sorensen_dice bray_curtis bray_curtis_faith bray_curtis_magurran canberra chisq chord euclidean gower hellinger kulczynski manhattan morisita_horn pearson soergel spearman_approx specprof	ANOVA G-test Paired *t* test Longitudinal correlation two sample *t* test adonis ANOSIM BEST Moran's I MRPP PERMANOVA PERMDISP db-RDA Mantel test
	*Phylogeny based metrics *	*Phylogeny based metrics *	
	PD whole tree	unifrac unifrac_g unifrac_g_full_tree unweighted_unifrac unweighted_unifrac_full_tree weighted_normalized_unifrac weighted_unifrac	

*Alpha diversity metrics for both the tools; ^§^beta diversity available metrics; ^†^parametric and non-parametric statistical tests available by default in the two tools.

**Table 3:** Summary of the main features of MG-RAST and QIIME.

	Availability	Analysis time	Prefiltering quality	16S rRNA detection mode	Taxonomic assignment methods*	Diversity analysis metrics^§^	Machine learning	Ease-of-use
MG-RAST	Web based	10 days (for private use)	Yes	Alignment with BLAT	One	One	No	Very simple GUI
QIIME	Native code plus additional applications	2 hours	Yes	OTU-picking	Several	Several	Yes	Requires basic informatic skills No GUI

GUI: Graphical user interface; *see Table 1; ^§^see Table 2.
